# Contributions of the Food and Nutrition Surveillance System (SISVAN) to the analysis of the nutritional profile of the Brazilian population: potentials and limitations

**DOI:** 10.1590/S2237-962220230004000001.EN

**Published:** 2023-12-08

**Authors:** Doroteia Aparecida Höfelmann, Cynthia Braga

**Affiliations:** 1 Universidade Federal do Paraná, Departamento de Nutrição, Curitiba, PR, Brazil; 2 Fundação Oswaldo Cruz, Instituto Aggeu Magalhães, Recife, PE, Brazil

Nutritional surveillance and dietary guidelines are integral components of the scope of the Brazilian National Health System (Sistema Único de Saúde - SUS), established in the Organic Health Law (Law No. 8,080, of September 19, 1990).^1^

The operationalization of a broad concept of food and nutrition surveillance requires the use of strategies to access and motivate different sources of information, such as population-based surveys, nutritional calls and scientific production, with an emphasis on monitoring the actions carried out by health services.^2^

In Brazil, the Food and Nutrition Surveillance System (Sistema de Vigilância Alimentar e Nutricional - SISVAN), created in the 1990s,^2^ has been one of the tools of the SUS used for collecting and consolidating information on the nutritional status and dietary habits of the population attending primary healthcare services in the country.^3^

One of the actions that contributed to expanding the System’s coverage was its integration into welfare programs. Early initiatives, in this regard, included the establishment of food and nutrition surveillance actions as prerequisites for the financing and implementation of welfare programs, with a focus on addressing malnutrition and providing care for children and pregnant women at nutritional risk. In the context of this strategy, food and nutrition surveillance actions were linked to the conditionalities of income transfer programs, such as Bolsa Família program, contributing to expanding access to primary healthcare services for socially vulnerable populations, aiming to ensure equity, which is one of the SUS guidelines.^2^

SISVAN data enable the acquisition of nutritional indicators for vulnerable groups, which are often underrepresented in population-based surveys, such as the indigenous population.^4^ Despite the advances in the System’s coverage, challenges persist in the most difficult areas to access in the national territory, in addition to regional inequalities.

Throughout its trajectory, the Epidemiology and Health Services (*Epidemiologia e Serviços de Saúde: revista do SUS* - RESS) journal has contributed to disseminating information on food and nutrition surveillance through the publication of articles analyzing the coverage and trends in the distribution of SISVAN indicators for different age groups,^5^ from children^6^ ^),(^ ^7^ and adults^8^ to the elderly.^9^ In this issue of RESS, the authors of the article entitled *Anthropometric indicators in traditional peoples and communities in Brazil: analysis of individual records from the Food and Nutrition Surveillance System, 2019* describe the prevalence of underweight and obesity among individuals identified as traditional peoples and communities, based on individual data from SISVAN. The study included information from traditional peoples and communities such as riverside dwellers, geraizeiros, quilombolas, and, less frequently, babassu coconut breakers, faxinalenses and the Romani people, showing significant heterogeneity in the distribution of nutritional conditions, including short stature and obesity, among different communities.^10^

The analysis of SISVAN data regarding the coverage and specific characteristics of each group is crucial for expanding knowledge of nutritional data generated from services. In addition to the advances in System coverage, the importance of strategies aimed at improving the collection of data related to food consumption markers stands out.^11^ However, challenges persist in the completion and, especially, the use of data generated by SISVAN for planning, management and evaluation of nutrition services in the SUS primary healthcare.^10^

Results of the analyses of the nutritional status of the study populations, based on data from the SISVAN, are relevant not only for revealing heterogeneity in the distribution of the various forms of malnutrition among these groups. They also call for public health responses that take into account the contexts and particularities of different peoples and communities in the country, ensuring food and nutritional security for the entire Brazilian population.
